# A frameshift variation in the *DSP* gene causes a novel subtype of atypical epidermolytic palmoplantar keratoderma: Case report

**DOI:** 10.3389/fmed.2025.1728762

**Published:** 2026-01-12

**Authors:** Chunli Lin, Huaqing Chen, Shuqin Lai, Shan Huang, Zimeng Guo, Lang Xie, Wei Zheng, Jingfa Lu, Zhaolin Zeng, Chunlei Wan, Longnian Li

**Affiliations:** 1 Department of Dermatology, First Affiliated Hospital of Gannan Medical University, Joint Organization of Jiangxi Clinical Medicine Research Center for Dermatology, Ganzhou, Jiangxi Province, China; 2 Department of Laser and Cosmetic Dermatology, Ganzhou Dermatosis Hospital, Ganzhou, Jiangxi Province, China; 3 The First Clinical Medical College, Gannan Medical University, Ganzhou, Jiangxi Province, China

**Keywords:** celladhesion, desmoplakin, epidermolyticpalmoplantar keratoderma, frameshiftvariation, phenotype

## Abstract

Palmoplantar keratoderma (PPK) represents a heterogeneous group of disorders characterized by hyperkeratosis of the palms and soles. Epidermolytic palmoplantar keratoderma (EPPK) is typically caused by variations in *KRT9* or *KRT1* genes. However, growing evidence suggests that defects in desmosomal genes, particularly desmoplakin (*DSP*), may underlie atypical variants. We report a 17-year-old girl with a 10-year history of yellowish, hyperkeratotic plaques with greasy scales on the dorsal hands, soles, and axillae. Histopathology revealed hyperkeratosis, parakeratosis, and acantholysis. Whole-exome sequencing (WES) identified a novel heterozygous frameshift variation in *DSP* (c.6218_6219dup, p. Ala2074Ter), confirmed by Sanger sequencing. This is the first report of an atypical EPPK caused by a *DSP* frameshift variation in the C-terminal domain, expanding the genotypic and phenotypic spectrum of PPK. The variant was absent from the gnomAD database. Functional studies demonstrated significant downregulation of adhesion molecules (*CDH1*, *JUP*, and *CTNNA1*) upon *DSP* knockdown, suggesting impaired desmosome-keratin anchoring as the pathogenic mechanism. This case reveals that *DSP* C-terminal domain variations can cause a new subtype of EPPK, providing new insights into PPK diagnosis and treatment.

## 1 Introduction

Palmoplantar keratoderma (PPK) encompasses a group of inherited skin disorders marked by hyperkeratosis in the palms and soles, with notable clinical and genetic heterogeneity (1). The structural integrity of the epidermis, particularly in high-stress areas such as palms and soles, is maintained by specialized intercellular junctions (2). Desmosome is classified as a calcium-dependent anchoring junction that tethers cells together through its extracellular contacts and internally links to the intermediate filament (IF) cytoskeleton. Through this linkage between cells, the desmosome provides tissues with the ability to resist mechanical forces (3). Desmoplakin (DSP), a core desmosomal protein, links keratin intermediate filaments to the cell membrane, maintaining epidermal integrity (2). Inherited desmosomal disease can lead to both cutaneous and cardiac disease, including certain forms of PPK (4).

Individual PPK cases can exhibit significant heterogeneity in clinical presentation and associated symptoms. Since various forms of hereditary PPK, like many other monogenic disorders, demonstrate very low prevalence rates, establishing a correct diagnosis remains challenging and often requires molecular genetic analysis (1). Advances in high-throughput sequencing have identified over 50 genes associated with PPK, including those encoding keratins (e.g., *KRT1* and *KRT9*), desmosomal proteins (e.g., *DSP*, *JUP*, and *DSG1*), and epidermal differentiation molecules (e.g., *SLURP1* and *CSTA*) (5). Epidermolytic palmoplantar keratoderma (EPPK) is one phenotype of PPK. The incidence rate of EPPK is approximately 2.2 to 4.4 cases per 100,000 live births (6). Classic EPPK, primarily caused by *KRT9* variations, manifests in infancy. The condition presents in adulthood with symmetric, presents with diffuse, yellowish hyperkeratosis on the palms and soles with an erythematous margin. A history of blistering may be present, and knuckle pads have been reported (7). Histologically characterized by vacuolar degeneration of the granular layer and keratin aggregation (6, 7). However, some PPK cases exhibit atypical clinical and histopathological features, implying unrecognized pathogenic mechanisms.

*DSP* is increasingly recognized in atypical PPK phenotypes. Recent systematic reviews highlight that *DSP* variations not only cause cutaneous manifestations but also give rise to a spectrum of additional syndromes. This spectrum includes severe syndromes such as Carvajal syndrome (characterized by striate PPK, woolly hair, and arrhythmogenic cardiomyopathy, CS) and skin fragility-woolly hair syndrome. The global epidemiology of *DSP*-related keratoderma is not fully established due to its rarity, but it is considered very rare, with sporadic cases reported worldwide. Recognized geographical clusters exist, such as in Greece (Naxos disease) and Ecuador (CS), yet cases have been identified across diverse populations, underscoring its universal occurrence. Critically, *DSP* pathogenic variations frequently confer a significant risk of multi-system involvement, particularly arrhythmogenic cardiomyopathy (ACM) (8, 9). The combination of PPK and hair shaft anomalies (e.g., woolly hair, WH) is a recognized warning signal for ACM, with 80.1% of such cases showing cardiac involvement (4). This link mandates a multidisciplinary management approach, including cardiac screening for affected individuals. Here, we describe a novel *DSP* frameshift variation (c.6218_6219dup) (RefSeq accession number: NM_004415.4, which corresponds to the GRCh37 p.13 or GRCh38 p.14 human reference genome assembly) in a patient with a novel phenotype of EPPK, featuring non-palmoplantar involvement (dorsal hands, axillae) and the presence of acantholysis on histology. *DSP* gene knockdown disrupted the expression of key adhesion molecules beyond desmosomes, including CDH1 and CTNNA1 (adherens junctions) (10) and JUP (bridging desmosomes and adherens junctions) (11). This reveals the interconnectedness and functional compensation among the diverse intercellular junctions (desmosomes, adherens, tight, gap) in keratinocytes. Desmosomal impairment thus propagates dysfunction through this molecular network, critically contributing to the adhesion defects observed.

## 2 Case report

**Figure 1:**
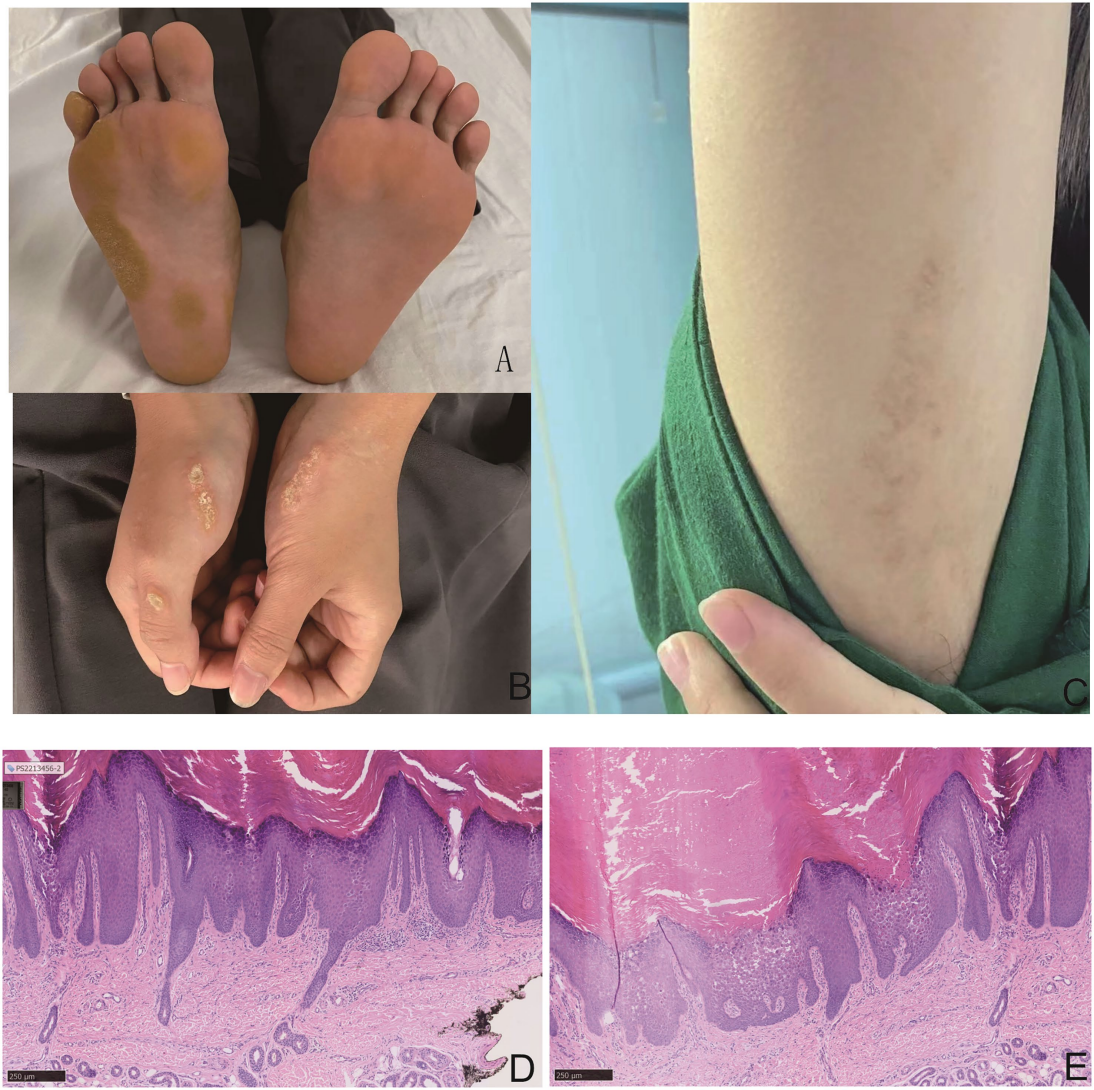
Clinical and histopathological features: yellow scaly keratotic plaques with oily appearance on the dorsum and soles of both hands; tan keratotic papules in the axilla **(A–C)**. Pathological biopsy: “grain” in stratum corneum and characteristic “round body” in the acantholytic area, separated by normal intervals **(D,E)**.

A 17-year-old girl presented with a 10-year history of yellowish, scaly, hyperkeratotic plaques on the dorsal hands, soles, and axillae (Figures 1A–C). Ten years ago, the patient first developed erythematous patches on the plantar surface; this was soon followed by mild skin thickening. At that time, the patient did not experience pruritus or other complaints that warranted medical attention. Subsequently, the area of the plantar surface erythema gradually expanded, the thickening worsened, and the skin became yellowish, scaly, rough, and hardened (presenting as hyperkeratotic plaques). Over the past decade, this skin condition (similar erythematous thickening, yellowish discoloration, scaling, and hardening) has progressively extended to involve the axillae and dorsal hands. The affected skin occasionally develops fissures (cracks), particularly when they occur on the plantar surface. Hematological, cardiac, pulmonary, and other examinations showed no significant abnormalities. Cardiac evaluation via electrocardiogram (ECG) revealed no abnormalities (Supplementary Figure 1). No abnormalities were detected in nails, hair, or teeth, and no friction-related lesions either. Other family members were asymptomatic. All procedures performed in studies involving human participants were in accordance with the ethical standards of the institutional and/or national research committee and with the 1964 Helsinki declaration and its later amendments or comparable ethical standards. After the patient signed the informed consent was obtained for a skin biopsy, we performed a skin pathological biopsy on the patient. Skin biopsy showed hyperkeratosis, parakeratosis, vacuolar degeneration of the granular layer, and acantholysis, which conform to a typical pathological manifestation of EPPK (Figures 1D,E).

**Figure 2:**
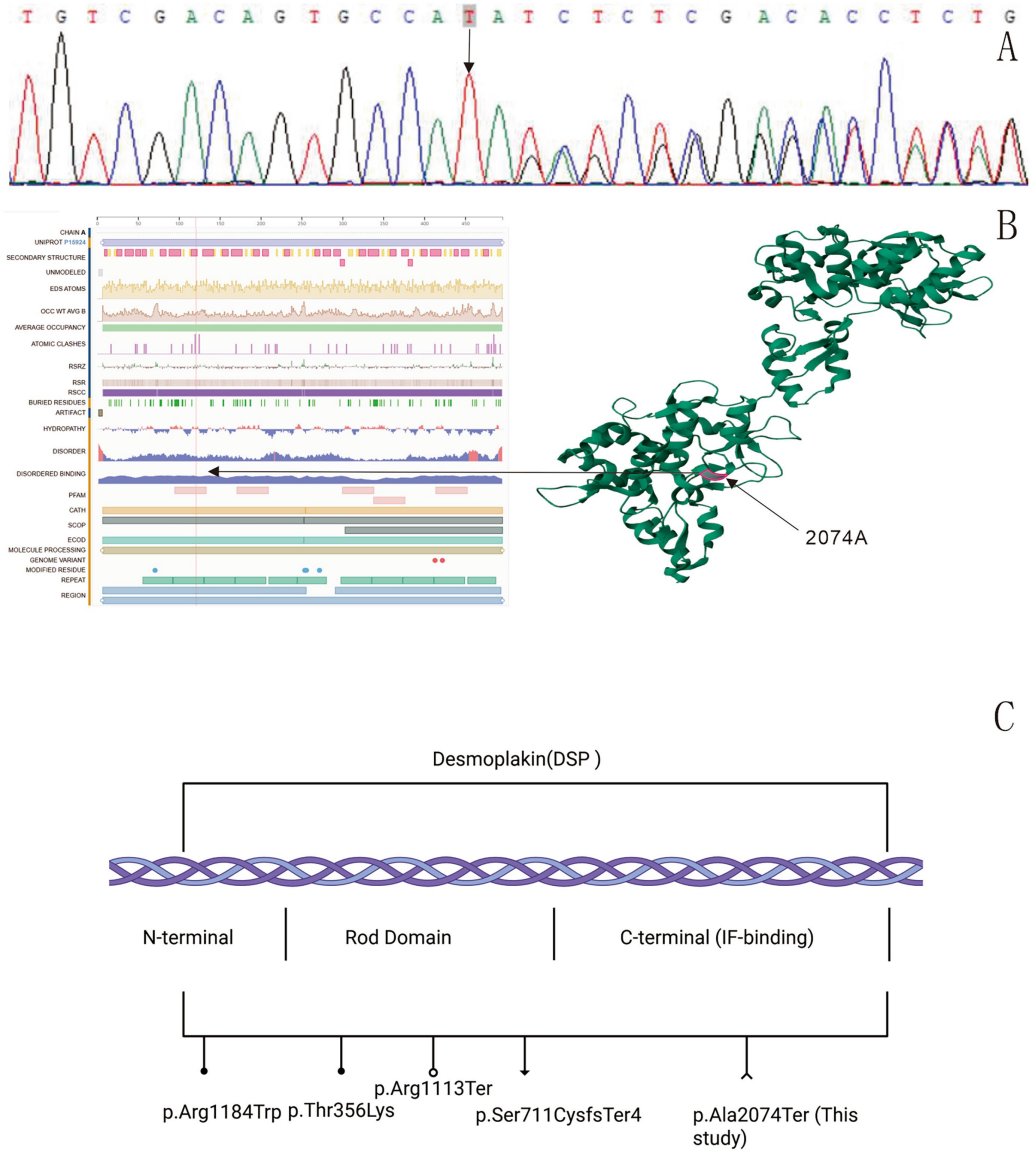
Sequencing results: a *DSP* variation c.6218_6,219 dup (p.Ala2074Ter) was found in the patient **(A)**. Protein structure model: the black arrow indicates the variation sequence (https://www.uniprot.org/uniprotkb/P15924/entry). The red highlighted region is the location of desmoplakin p.Ala2074Ter. A TA duplication between nucleotides 6,218 and 6,219 in the coding region of the *DSP* gene, resulting in a frameshift variation (p.Ala2074Ter). This variation introduces a premature termination codon at the first downstream stop signal, leading to the complete loss of all subsequent amino acids **(B)**. Schematic representation of reported *DSP* mutations. ●: Denote missense mutations; ○: denote nonsense mutations; ▼: denote frameshift mutations. ∧: The novel nonsense mutation (p.Ala2074Ter) reported in this study **(C)**.

Whole-exome sequencing (WES) identified a heterozygous *DSP* variation (c.6218_6219dup), which was verified by Sanger sequencing (Figure 2A). This TA duplication causes a frameshift p. Ala2074Ter leading to premature termination (Figures 2B,C), representing a novel variant absent in gnomAD. Parental genetic testing was not performed as the patient’s parents and sister declined to undergo genetic analysis. According to the ACMG-AMP guidelines, the variant was classified as likely pathogenic, meeting the following criteria: PVS1 (null variant in a gene where loss-of-function is a known mechanism of disease), PM2 (absent from population databases), and PS3 (*in vitro* functional studies supportive of a damaging effect) (12).

**Figure 3:**
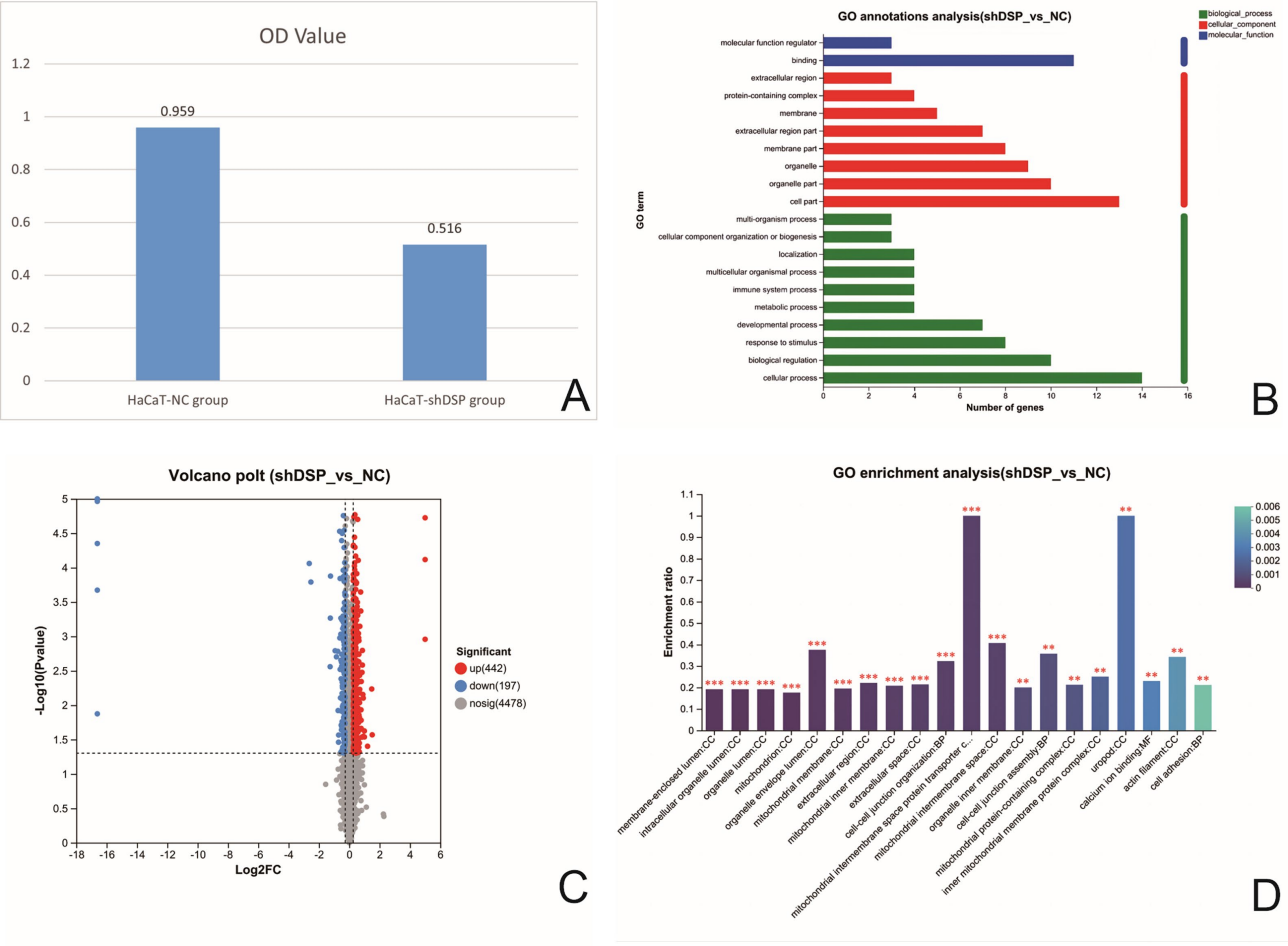
Comparison of cell adhesion rates of HaCaT cells after *DSP* interference **(A)**. Histogram of GO classification statistics for differential genes after *DSP* gene interference. (Note: In the figure, the vertical coordinate represents the second-level classification term of GO, the horizontal coordinate represents the number of genes compared to the second-level classification, and the three colors represent the three classifications) **(B)**. Volcano map of differential proteins in DSP interfered stable strain (the abscissa is the fold change of protein expression in the sh*DSP* group/NC group), and the ordinate is the statistical test value of the difference in protein expression, namely the *p*-value. The smaller the *p*-value, the more significant is the expression difference. Each dot in the figure represents a specific protein; the dots on the right are differentially upregulated proteins, and the dots on the left are differentially down-regulated proteins. The more to the left, the more significant the difference in expression is **(C)**. GO enrichment analysis of differentially expressed proteins in DSP-transfected strains (the abscissa represents enriched entries, and the ordinate represents enrichment rates). The column color gradient indicates the significance of enrichment, where a *P* or FDR value of less than 0.001 is marked as ***, less than 0.01 as **, and less than 0.05 as *) **(D)**.

To further investigate the association between *DSP* gene variations and EPPK, we successfully constructed a stable HaCaT cell line with *DSP* gene interference (HaCaT-h-*DSP*-shRNA-ZSGREEN-PURO) (Supplementary Table 1) using the lentiviral infection method. The ethics of this study has been approved by the Research Ethics Committee of the First Affiliated Hospital of Gannan Medical University (LLSC-2025374). *In vitro* cell experiments showed that compared with the negative control group (HaCaT/NC), the cell adhesion and proliferation abilities of *DSP*-knockdown HaCaT cells (HaCaT/sh*DSP*) were significantly weakened (*p* < 0.05) (Figure 3A). Transcriptomic profiling revealed 16 differentially expressed genes (DEGs) (Supplementary Table 2). Gene Ontology (GO) annotation indicated that these DEGs were primarily associated with cellular processes (biological process, BP), organelle components (cellular component, CC), and binding activities (molecular function, MF) (Figure 3B). Six DEGs—*cadherin 26 (CDH26)*, *S100 calcium-binding protein P (S100P)*, *GRAM domain containing 2A (GRAMD2A)*, *keratin 6B (KRT6B)*, *keratin 4 (KRT4)*, and *S100 calcium-binding protein A7 (S100A7)*—were screened as potentially relevant to the pathology of PPK and are implicated in calcium ion binding, cell adhesion, and epidermal development (Supplementary Table 3).

To further elucidate the underlying molecular pathways, a proteomic analysis was conducted. (The raw proteomics data have been deposited in the iProX database under the accession number PXD070791). Proteomic analysis identified 639 differentially expressed proteins (DEPs) (Figure 3C and Supplementary Table 4). GO enrichment analysis demonstrated significant enrichment of these DEPs in terms related to cell adhesion, calcium ion binding, and the catenin complex (Figure 3D). Three target protein sets were constructed based on these functional categories (Supplementary Figure 2). Cross-analysis among these sets identified 10 key DEPs: EGF like repeats and discoidin domains 3 (EDIL3), thrombospondin 1 (THBS1), desmoglein 2 (DSG2), desmoglein 1 (DSG1), cadherin 2 (CDH2), cadherin 1 (CDH1), cadherin 13 (CDH13), junction plakoglobin (JUP), catenin delta 1 (CTNND1), and catenin alpha 1 (CTNNA1) (Supplementary Table 5). After DSP knockdown, the expression levels of molecules related to cell adhesion, such as E-cadherin (CDH1) (adherens junctions), catenins (CTNND1, CTNNA1) (adherens junctions), JUP (bridging desmosomes and adherens junctions), and calcium-binding protein (S100A7), significantly decreased (*p* < 0.05).

## 3 Discussion

PPK represents a group of dermatoses with high clinical and genetic heterogeneity. Different genetic variations can lead to varying clinical manifestations of EPPK (1). Table 1 shows the clinical features, gender, and age of EPPK caused by different variation sites in the *DSP* gene (Table 1). Our case presented a diagnostic challenge due to its combination of epidermolytic hyperkeratosis, acantholysis, and a non-palmoplantar distribution. This necessitates a careful differentiation from classic forms of PPK and other acantholytic disorders. Classic *KRT9*-related EPPK is characterized by diffuse, waxy, yellowish hyperkeratosis strictly localized to the pressure points of the palms and soles, and histology shows vacuolar degeneration without prominent acantholysis. The pathogenesis involves impaired keratin dimer assembly and reduced mechanical stability (6, 7). In contrast, our proband demonstrated extensive lesions involving the dorsal hands and axillae, a finding not seen in classic EPPK. While DSP dysfunction theoretically affects all keratinocytes, phenotypic expression can be modulated by regional factors. The extensive involvement in our case may be attributed to the particular susceptibility of flexural and dorsal skin, which experiences constant mechanical stress from stretching and friction. The underlying cause of this susceptibility is likely due to disrupted desmosome-keratin anchoring from the DSP C-terminal truncation (4). Furthermore, our case is distinct from focal or striate PPK. While those forms present with localized or linear keratoderma. More importantly, the hallmark histologic features of epidermolysis and acantholysis observed in our case are not characteristic of typical focal or striate PPK (13). Alternatively, we acknowledge that the precise reason for this specific distribution cannot be fully explained by our current data and may involve yet unidentified modifying factors.

**Table 1:** Additional symptoms of EPPK with *DSP* gene variations.

Variation site	Variation protein	Age (years)	Gender	Variation	Reported number of cases	References
3550C > T	Arg1184Trp	61	Male	Focal PPK and WH	1	Xue K, 2019 (20)
1067C > A	Thr356Lys	14	Male	WH, dilated cardiomyopathy (DCM) with severe left ventricular insufficiency	3	Pigors M, 2015 (21)
7566_7567delinsC	Arg2522SerfsTer39	5	Male	Striate and focal keratoderma, keratotic papules		
2131_2132del	Ser711CysfsTer4	10	Female	Mild focal plantar keratoses and hypotrichosis		
2493del	Glu831AspfsTer33	23–66	Seven females and two males	Focal PPK and ACM	9	Karvonen V, 2022 (22)
3337C > T	Arg1113Ter	40	Four females and five Males	Skin fragility, WH, PPK, ACM and, DCM	1	Andrei D, 2024 (23)
6310del	Thr2104GlnfsTer12	30–69	Males	Curly hair, PPK, and ACM with increased trabeculation	9	Krista Heliö, 2023 (24)
6687del	Arg2229SerfsTer32	21–80	Five females and one male	Mild PPK and non-lethal cardiomyopathy	6	Vermeer MCSC, 2022 (25)
6687delA/273 + 5G > A	Predicted to modify the exon 2 splice site	23–52	Two females	Cardiomyopathy, PPK and WH	2	
4198C > T	Arg1400Ter	3	Male	Cardiomyopathy congenital alopecia, nail dystrophy, PPK, and follicular hyperkeratosis with plugging on the extensor surfaces	1	Antonov NK, 2015 (26)
6850C > T	Arg2284Ter					

Our patient lacked cardiac symptoms or hair shaft anomalies; prior studies emphasize that *DSP* variations—especially truncating variants—are strongly associated with arrhythmogenic cardiomyopathy. For example, Carvajal syndrome (N-terminal mutations) shows PPK, cardiomyopathy, and WH (absent in our case), while skin fragility-WH syndrome (rod domain mutations) causes blistering and hair anomalies (versus predominant hyperkeratosis here). Notably, cardiac involvement may emerge later in life; thus, lifelong cardiac monitoring (e.g., echocardiography, ECG) is recommended for all *DSP* variation carriers, regardless of current cardiac status.

Unlike Darier disease (DD), which results from *ATP2A2* variations encoding SERCA2, it shares the histologic finding of acantholysis. The DD phenotype typically manifests during adolescence or early adulthood with greasy, hyperkeratotic papules in seborrheic areas (central chest, back, scalp margins, and flexures). The disease is characterized by distinctive nail abnormalities and, in severe forms, extensive malodorous plaques that impair mobility. Neurological and psychiatric abnormalities have been reported in DD. Histopathologically, DD is defined by suprabasal acantholysis leading to lacunae, along with the presence of “corps ronds” and “grains” (14). Our case had *DSP* (not *ATP2A2*) variations, palmoplantar involvement (unusual in DD), and cell adhesion (not calcium signaling) defects (15). This structured comparison highlights the novelty of our case, which fits neither the classic EPPK nor the DD profile, instead occupying a unique position in the PPK spectrum.

DD-like phenotypes (as in our case) suggest that C-terminal *DSP* variations may induce unique acantholytic hyperkeratosis. The wild-type DSP protein consists of 2,871 amino acids (UniProt P15924). Sanger sequencing of the patient revealed a TA duplication between nucleotides 6,218 and 6,219 in the coding region of the *DSP* gene, resulting in a frameshift variation (p. Ala2074Ter). This variation introduces a premature termination codon at the first downstream stop signal, leading to the complete loss of all subsequent amino acids. Consequently, the entire C-terminal intermediate filament (IF)-binding domain is truncated. The truncated DSP loses its ability to anchor desmosomes to keratin intermediate filaments, causing intercellular adhesion defects in keratinocytes. This disrupts mechanical stress transmission between cells, resulting in epidermal fragility with blister formation and acantholysis (5). We propose a pathogenic pathway: The truncated DSP impairs keratin filament anchoring, leading to mechanical stress imbalance, which causes acantholysis and downregulation of adhesion molecules (e.g., E-cadherin), resulting in aberrant differentiation (16); this subsequently downregulates S100A7, mediating proliferation and differentiation defects (17).

We identified a novel heterozygous *DSP* frameshift variation (c.6218_6219dup, p. Ala2074Ter) in a novel subtype of atypical EPPK. A limitation of this study is the inability to perform parental genetic testing to definitively determine the mode of inheritance (*de novo* vs. inherited), as family members declined testing. Nonetheless, the variant was classified as likely pathogenic according to ACMG criteria. The variation is predicted to result in a truncated protein lacking the C-terminal intermediate filament-binding domain, which is critical for anchoring keratin to the desmosomal plaque. Our functional studies support the pathogenicity of this variant. *DSP* knockdown in keratinocytes significantly impaired cell adhesion and proliferation. Multi-omics analyses revealed that *DSP* deficiency led to widespread downregulation of key adhesion molecules. While these proteomic findings provide crucial mechanistic insight by confirming the disruption of keratinocyte adhesion pathways and validating the variant’s pathogenicity, they do not directly alter the immediate clinical management strategy for this patient beyond reinforcing the need for cardiac surveillance, which is already standard of care for *DSP*-related disease.

This study is significant as the first report of atypical EPPK from a *DSP* C-terminal domain frameshift variation, expanding both the *DSP* variation spectrum and phenotypic range. Mechanistic insights suggest potential therapies such as topical retinoids (18) or JAK inhibitors (19). After unremarkable preoperative clearance, the patient had the hand lesions removed by excision of bilateral superficial hand masses with concurrent random-pattern flap reconstruction, and was discharged safely. The unresolved implications for cardiac desmosomes warrant long-term follow-up.

## 4 Conclusion

We describe a novel atypical EPPK caused by *DSP* c.6218_6219dup (p. Ala2074Ter), with atypical clinicopathological features. Functional studies demonstrate that *DSP* deficiency disrupts epidermal homeostasis through widespread adhesion molecule dysregulation, offering new perspectives for PPK diagnosis and treatment.

## Data Availability

The original contributions presented in the study are included in the article/Supplementary material, further inquiries can be directed to the corresponding author.

## References

[1] Schiller S Seebode C Hennies HC Giehl K Emmert S. Palmoplantar keratoderma (PPK): acquired and genetic causes of a not so rare disease. J Dtsch Dermatol Ges. (2014) 12:781–8. doi: 10.1111/ddg.12418, 25176457

[2] Green KJ Jaiganesh A Broussard JA. Desmosomes: essential contributors to an integrated intercellular junction network. F1000Res. (2019) 8:2150. doi: 10.12688/f1000research.20942.1, 31942240 PMC6944264

[3] Najor NA. Desmosomes in human disease. Annu Rev Pathol. (2018) 13:51–70. doi: 10.1146/annurev-pathol-020117-044030, 29414250

[4] Polivka L Bodemer C Hadj-Rabia S. Combination of palmoplantar keratoderma and hair shaft anomalies, the warning signal of severe arrhythmogenic cardiomyopathy: a systematic review on genetic desmosomal diseases. J Med Genet. (2016) 53:289–95. doi: 10.1136/jmedgenet-2015-103403, 26399581

[5] Samuelov L Sprecher E. Inherited desmosomal disorders. Cell Tissue Res. (2015) 360:457–75. doi: 10.1007/s00441-014-2062-y, 25487406

[6] Li Y Tang L Han Y Zheng L Zhen Q Yang S . Genetic analysis of KRT9 gene revealed previously known mutations and genotype-phenotype correlations in Epidermolytic palmoplantar keratoderma. Front Genet. (2019) 9:645. doi: 10.3389/fgene.2018.00645, 30666268 PMC6330350

[7] Liu X Qiu C He R Zhang Y Zhao Y. *Keratin 9* L164P mutation in a Chinese pedigree with epidermolytic palmoplantar keratoderma, cytokeratin analysis, and literature review. Mol Genet Genomic Med. (2019) 7:e977. doi: 10.1002/mgg3.977, 31525823 PMC6825865

[8] Yuan ZY Cheng LT Wang ZF Wu YQ. Desmoplakin and clinical manifestations of desmoplakin cardiomyopathy. Chin Med J. (2021) 134:1771–9. doi: 10.1097/CM9.0000000000001581, 34343150 PMC8367056

[9] Protonotarios N Tsatsopoulou A. Naxos disease: cardiocutaneous syndrome due to cell adhesion defect. Orphanet J Rare Dis. (2006) 1:4. doi: 10.1186/1750-1172-1-4, 16722579 PMC1435994

[10] Campbell HK Maiers JL DeMali KA. Interplay between tight junctions & adherens junctions. Exp Cell Res. (2017) 358:39–44. doi: 10.1016/j.yexcr.2017.03.061, 28372972 PMC5544570

[11] Whittock NV Eady RA McGrath JA. Genomic organization and amplification of the human plakoglobin gene (JUP). Exp Dermatol. (2000) 9:323–6. doi: 10.1034/j.1600-0625.2000.009005323.x, 11016852

[12] Richards S Aziz N Bale S Bick D Das S Gastier-Foster J . Standards and guidelines for the interpretation of sequence variants: a joint consensus recommendation of the american college of medical genetics and genomics and the association for molecular pathology. Genet Med. (2015) 17:405–24. doi: 10.1038/gim.2015.30, 25741868 PMC4544753

[13] Braun-Falco M. Hereditary palmoplantar keratodermas. J Dtsch Dermatol Ges. (2009) 7:971–84. doi: 10.1111/j.1610-0387.2009.07058.x, 19341430

[14] Zheng L Jiang H Mei Q Chen B. Identification of two novel Darier disease-associated mutations in the ATP2A2 gene. Mol Med Rep. (2015) 12:1845–9. doi: 10.3892/mmr.2015.3605, 25872913 PMC4464092

[15] Cabral RM Tattersall D Patel V McPhail GD Hatzimasoura E Abrams DJ . The DSPII splice variant is critical for desmosome-mediated HaCaT keratinocyte adhesion. J Cell Sci. (2012) 125:jcs.084152. doi: 10.1242/jcs.084152 22454510

[16] Wong SHM Fang CM Chuah LH Leong CO Ngai SC. E-cadherin: its dysregulation in carcinogenesis and clinical implications. Crit Rev Oncol Hematol. (2018) 121:11–22. doi: 10.1016/j.critrevonc.2017.11.010, 29279096

[17] Eckert RL Broome AM Ruse M Robinson N Ryan D Lee K. S100 proteins in the epidermis. J Invest Dermatol. (2004) 123:23–33. doi: 10.1111/j.0022-202X.2004.22719.x, 15191538

[18] Marukian NV Levinsohn JL Craiglow BG Milstone LM Choate KA. Palmoplantar Keratoderma in Costello Syndrome Responsive to Acitretin. Pediatr Dermatol. (2017) 34:160–2. doi: 10.1111/pde.13057, 28008647

[19] Sanchez GAM Reinhardt A Ramsey S Wittkowski H Hashkes PJ Berkun Y . JAK1/2 inhibition with baricitinib in the treatment of autoinflammatory interferonopathies. J Clin Invest. (2018) 128:3041–52. doi: 10.1172/JCI98814, 29649002 PMC6026004

[20] Xue K Zheng Y Cui Y. A novel heterozygous missense mutation of *DSP* in a Chinese Han pedigree with palmoplantar keratoderma. J Cosmet Dermatol. (2019) 18:371–6. doi: 10.1111/jocd.12533, 29607617

[21] Pigors M Schwieger-Briel A Cosgarea R Diaconeasa A Bruckner-Tuderman L Fleck T . Desmoplakin mutations with palmoplantar keratoderma, woolly hair and cardiomyopathy. Acta Derm Venerol. (2015) 95:337–40. doi: 10.2340/00015555-1974, 25227139

[22] Karvonen V Harjama L Heliö K Kettunen K Elomaa O Koskenvuo JW . A novel desmoplakin mutation causes dilated cardiomyopathy with palmoplantar keratoderma as an early clinical sign. Acad Dermatol Venereol. (2022) 36:1349–58. doi: 10.1111/jdv.18164, 35445468 PMC9545885

[23] Andrei D Bremer J Kramer D Nijenhuis AM Van Der Molen M Diercks GFH . Epidermal growth factor receptor inhibition leads to cellular phenotype correction of DSP -mutated keratinocytes. Exp Dermatol. (2024) 33:e15046. doi: 10.1111/exd.15046, 38509711

[24] Heliö K Brandt E Vaara S Weckström S Harjama L Kandolin R . DSP c.6310delA p.(Thr2104Glnfs*12) associates with arrhythmogenic cardiomyopathy, increased trabeculation, curly hair, and palmoplantar keratoderma. Front Cardiovasc Med. (2023) 10:1130903. doi: 10.3389/fcvm.2023.1130903, 37008330 PMC10050721

[25] Vermeer MCSC Andrei D Kramer D Nijenhuis AM Hoedemaekers YM Westers H . Functional investigation of two simultaneous or separately segregating *DSP* variants within a single family supports the theory of a dose-dependent disease severity. Exp Dermatol. (2022) 31:970–9. doi: 10.1111/exd.14571, 35325485 PMC9322008

[26] Antonov NK Kingsbery MY Rohena LO Lee TM Christiano A Garzon MC . Early-onset heart failure, alopecia, and cutaneous abnormalities associated with a novel compound heterozygous mutation in Desmoplakin. Pediatr Dermatol. (2015) 32:102–8. doi: 10.1111/pde.12484, 25516398

