# Supplementary material for: A frameshift variation in the DSP gene causes a novel subtype of atypical epidermolytic palmoplantar keratoderma: Case report
Source: Front Med (Lausanne). 2026 Jan 12;12:1728762. doi: 10.3389/fmed.2025.1728762 (PMC12832874; doi:10.3389/fmed.2025.1728762)
Supplement: Supplementary file 1 [file Data_Sheet_1.docx]

Supplementary Material

# Supplementary Figures and Tables

## 1.Supplementary Figures


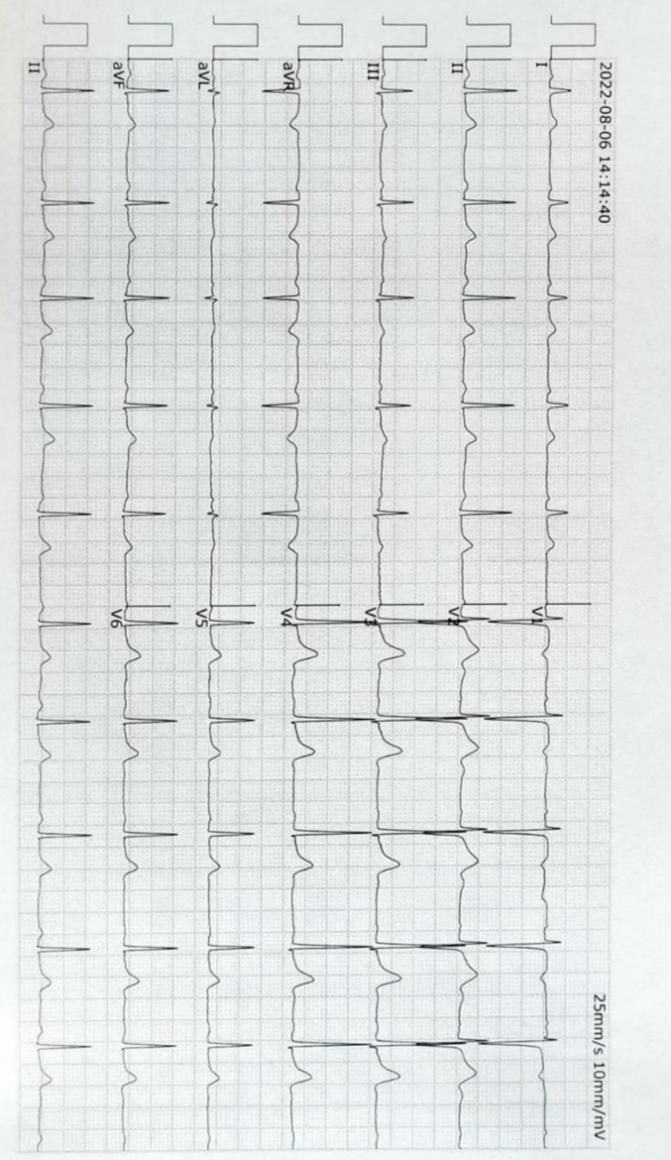


Supplementary Figure 1 ECG Results:Ventricular rate: 61 bpm; QRS axis: 65; P wave duration: 104 ms; QT/QTc interval: 427/430 ms; PR interval: 167 ms; RV5/SV1: 1.02/1.34 mV; QRS duration: 93 ms; RV5 + SV1: 2.36 mV; Interpretation: Normal ECG.


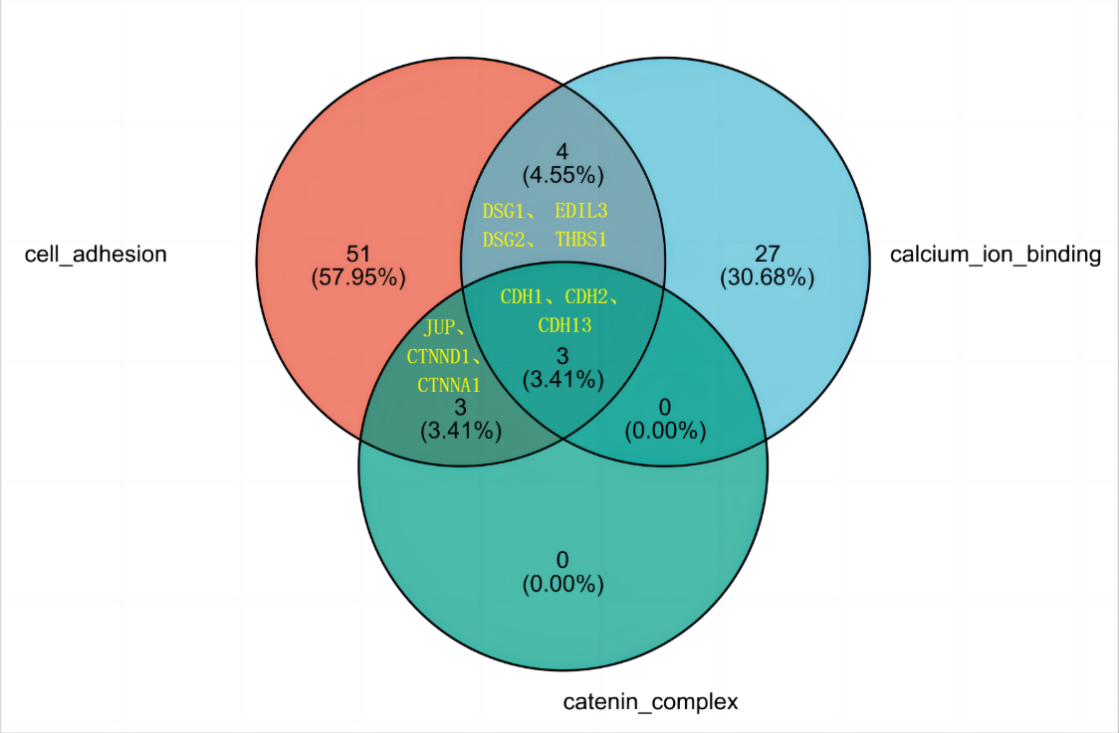


Supplementary Figure 2 Venn diagram of the three target protein sets. Note: In the figure, the overlapping part represents the number of differential proteins shared by multiple difference groups, and the yellow font is the name of differential proteins. The part without overlap represents the number of differential proteins unique to the difference group, and the number represents the corresponding number of proteins.

## 2.Supplementary Table

## Supplementary Table 1 Primer synthesis sequences of the target gene (The RNA interference vector is pHBLV-U6-MCS-CMV-ZsGreen-PGK-PURO).

| Type | Name | Sequence |
| --- | --- | --- |
| Control viral vector | **siRNA** | TTCTCCGAACGTGTCACGTAA |
|  | **shRNA** | Top strand: GATCCGTTCTCCGAACGTGTCACGTAATTCAAGAGATTACGTGACACGTTCGGAGAATTTTTTC  Bottom strand: AATTGAAAAAATTCTCCGAACGTGTCACGTAATCTCTTGAATTACGTGACACGTTCGGAGAACG |
| Target gene | **siRNA** | AGCAAATGCGAGCCCTTTATA |
|  | **shRNA** | Top strand: GATCCGAGCAAATGCGAGCCCTTTATACTCGAGTATAAAGGGCTCGCATTTGCTTTTTTTG  Bottom strand: AATTCAAAAAAAGCAAATGCGAGCCCTTTATACTCGAGTATAAAGGGCTCGCATTTGCTCG |

Supplementary Table 2 Statistical table of differentially expressed genes between *shDSP* group and NC group.

| Gene Name | Gene Description | Expression Trend |
| --- | --- | --- |
| *SEMA5B* | Semaphorin 5B | up |
| *ATP6V1B1* | ATPase H+ Transporting V1 Subunit B1 | up |
| *CXCL14* | C-X-C Motif Chemokine Ligand 14 | up |
| *SCGB1A1* | Secretoglobin Family 1A Member 1 | up |
| *S100P* | S100 Calcium Binding Protein P | up |
| *KRT4* | Keratin 4 | up |
| *KRT6B* | Keratin 6B | up |
| *GRAMD2A* | GRAM Domain Containing 2A | down |
| - |  | up |
| *TMEM238* | Transmembrane Protein 238 | down |
| - |  | down |
| - |  | down |
| *H4C14* | H4 Clustered Histone 14 | down |
| *CDH26* | Cadherin 26 | down |
| *S100A7* | S100 Calcium Binding Protein A7 | down |
| *EEF1A2* | Eukaryotic Translation Elongation Factor 1 Alpha | down |

Note: "-" indicates that this differentially expressed gene currently lacks clear annotation.

Supplementary Table 3 The differentially expressed target gene sets of *DSP* interference stable transformants were screened.

| Gene Name | GO Annotation | Expression Trend | Fold Change |
| --- | --- | --- | --- |
| *CDH26* | Cell adhesion; cadherin binding; calcium ion binding; catenin complex. | down | 0.23 |
| *S100P* | Calcium ion binding; cadherin binding; calcium-dependent protein binding. | up | 3.37 |
| *GRAMD2A* | Regulation of calcium storage. | up | 1.18 |
| *KRT6B* | Keratinization; structural constituent of cytoskeleton. | up | 3.29 |
| *KRT4* | Epithelial cell differentiation; negative regulation of epithelial cell proliferation; keratinization; intermediate filament cytoskeleton. | up | 27.03 |
| *S100A7* | Epidermis development; focal adhesion; calcium ion binding. | down | 0.48 |

Supplementary Table 4 Top 15 up- and down-regulated significantly altered proteins.

| Protein Name | Fold Change | Expression Trend | *P*-value |  |
| --- | --- | --- | --- | --- |
| TAGLN3 | 32 | up | 6.64×10^-5^ |  |
|  |  |  |  |  |
| DSG1 | 32 | up | 8.86×10^-6^ |  |
|  |  |  |  |  |
| AKAP12 | 32 | up | 1.10×10^-3^ |  |
|  |  |  |  |  |
| SLC35D1 | 2.85 | up | 0.03 |  |
|  |  |  |  |  |
| SELENON | 2.79 | up | 5.81×10^-3^ |  |
|  |  |  |  |  |
| EIF2B2 | 2.31 | up | 0.04 |  |
|  |  |  |  |  |
| RTN3 | 1.99 | up | 0.03 |  |
|  |  |  |  |  |
| CCNB1 | 1.9 | up | 0.03 |  |
|  |  |  |  |  |
| TOMM6 | 1.9 | up | 0.01 |  |
|  |  |  |  |  |
| DPAGT1 | 1.86 | up | 0.01 |  |
| CMC1 | 1.82 | up | 1.60×10^-3^ |  |
|  |  |  |  |  |
| STX3 | 1.79 | up | 3.33×10^-3^ |  |
|  |  |  |  |  |
| ERH | 1.73 | up | 4.52×10^-3^ |  |
|  |  |  |  |  |
| MRPS18C | 1.72 | up | 0.02 |  |
|  |  |  |  |  |
| IGFBP6 | 1.71 | up | 0.01 |  |
|  |  |  |  |  |
| AP3D1 | 1.00×10^-5^ | down | 4.90×10^-7^ |  |
|  |  |  |  |  |
| AP5B1 | 1.00×10^-5^ | down | 2.02×10^-4^ |  |
|  |  |  |  |  |
| ARHGEF39 | 1.00×10^-5^ | down | 0.01 |  |
|  |  |  |  |  |
| EPS8 | 1.00×10^-5^ | down | 2.81×10^-9^ |  |
|  |  |  |  |  |
| FLJ11193 | 1.00×10^-5^ | down | 3.46×10^-5^ |  |
|  |  |  |  |  |
| MTMR2 | 1.00×10^-5^ | down | 8.89×10^-7^ |  |
| FGD3 | 0.17 | down | 1.52×10-4 |  |
| PERP | 0.42 | down | 2.75×10-3 |  |

Supplementary Table 5 The expression levels of target proteins.

| Protein | Proteins | Trend |
| --- | --- | --- |
| EDIL3 | EGF Like Repeats And Discoidin Domains 3 | down |
| THBS1 | Thrombospondin 1 | up |
| DSG2 | Desmoglein 2 | down |
| DSG1 | Desmoglein 1 | up |
| CDH2 | Cadherin 2 | down |
| CDH1 | Cadherin 1 | down |
| CDH13 | Cadherin 3 | down |
| JUP | Junction Plakoglobin | down |
| CTNND1 | Catenin Delta 1 | down |
| CTNNA1 | Catenin Alpha 1 | down |

**Supplementary Methods**

**Part 1: A Case of Palmoplantar Keratoderma**

**1.1 Experimental Materials**

1.1.1 Study Subject

This study enrolled one patient with palmoplantar keratoderma (PPK) who presented to our department. The patient's clinical manifestations and histopathological features met the diagnostic criteria for PPK.

After providing informed consent and obtaining approval from our hospital's Ethics Committee, 5 mL of peripheral venous blood was drawn from the patient and her sister. The blood was immediately placed into EDTA anticoagulant tubes. If whole-exome sequencing could not be performed immediately, the samples were briefly stored at 4 C for up to approximately 4 hours. It is preferable to perform genetic testing promptly to prevent degradation of relevant components. Detailed clinical information was also collected, including name, age, gender, disease duration, comorbidities, lesion morphology and distribution, medical history, family history, results of auxiliary examinations, and histopathological findings.

1.1.2 Experimental Reagents

1.1.2.1 Reagents for Histopathological Examination

Ethanol, 10% formalin solution, Eosin reagent, Xylene reagent, Hematoxylin reagent, Paraffin wax.

1.1.3 Major Laboratory Instruments

| Instrument Name | Manufacturer | Model/Catalog Number |
| --- | --- | --- |
| Tissue Processor | Wuhan Junjie | JJ-12J |
| Paraffin Embedding Machine | Leica, Germany | Leica EG1150 |
| Microtome | Thermo | HM340 |
| Freezing Stage | Wuhan Junjie | JB-L5 |
| Tissue Spreader | Jinhua Kedi | KD-P |
| Slides & Cover Slips | Jiangsu Shitai | 10212432C |

## Experimental Methods

### 1.2.1 Preparation of Skin Histopathological Specimens

1). Biopsy Procedure: A skin biopsy was performed by a physician from our department. The biopsy steps included: localization, disinfection, anesthesia, excision, and suturing. The excised specimen was immediately placed in a pre-prepared 10% formalin solution and arranged for delivery to the Pathology Department of our hospital for further processing according to the following protocol.

2). Pathological Specimen Preparation Protocol: Fixation, rinsing and dehydration, clearing, wax infiltration, embedding, sectioning, staining, dehydration, clearing, and mounting.

1.2.2 Collection of Peripheral Blood Samples for Genetic Sequencing

1.2.2.1 Collection of Peripheral Blood from the Patient and Sister
After communication with the patient and her family members, and upon obtaining consent, 5 mL of peripheral venous blood was collected from the patient. The blood was placed in an EDTA-anticoagulant tube, mixed gently, and stored at -20°C.

2.2.2.2 DNA Extraction from Peripheral Blood
Genomic DNA was extracted from the patient's peripheral blood according to the following steps:

1). After thawing the frozen EDTA-anticoagulated blood sample at room temperature, 300 μL of the blood sample was transferred to a 1.5 mL microcentrifuge tube. 900 μL of cell lysis buffer was added, the mixture was incubated at room temperature for 10 minutes, mixed thoroughly, and then centrifuged at 13,000-16,000 rpm for 20 seconds. The supernatant was discarded.

2). 300 μL of nuclear lysis solution and 100 μL of protein precipitation solution were added to the pellet and mixed thoroughly.

3). The tube was centrifuged at 14,000 rpm for 3 minutes.

4). The supernatant was transferred to a new 1.5 mL microcentrifuge tube. 300 μL of isopropanol was added and mixed gently by inversion, resulting in the formation of white flocculent material.

5). The tube was centrifuged at 14,000 rpm for 1 minute, and the supernatant was discarded. 100 μL of 70% ethanol was then added and mixed gently.

6). The tube was centrifuged at 14,000 rpm for 1 minute, the supernatant was discarded, and the tube was inverted on filter paper to air dry for 15 minutes.

7). 100 μL of DNA resuspension buffer was added to dissolve the DNA, and the tube was placed at 4°C for 24 hours.

8). The DNA solution could be stored temporarily at 4°C or for long-term storage at -20°C.

1.2.2.3 Whole Exome Sequencing and Sanger Sequencing
Following DNA extraction from the samples, the long DNA chains were fragmented into pieces primarily ranging from 200-300 bp. After end repair and “A”-tailing of the DNA fragments, specific adapters were ligated. Finally, PCR amplification was performed to construct a whole-genome library, which was sequenced using the Illumina platform sequencer.

**2.Result**

**2.1 Pathological Biopsy**

Microscopic examination revealed: “grain” in stratum corneum and characteristic “round body” in acantholytic area, separated by normal intervals (manuscription Figure 1D-E)

**2.2 Whole Exome Sequencing and Sanger Sequencing Results**

Genetic sequencing identified a *DSP* gene variation in the patient, specifically *DSP* c.6218_6219dup (p. Ala2074Ter). This variation was confirmed by Sanger sequencing. This variant is a duplication of thymine and adenine (TA) between nucleotides 6218 and 6219 in the coding region, resulting in a frameshift mutation p. Ala2074Ter, which is predicted to lead to premature termination of translation. (manuscription Figure 2A)

**2.3 Conclusion**

The clinical and pathological features of this patient are consistent with the diagnosis of palmoplantar keratoderma (PPK). The *DSP* gene variant c.6218_6219dup (p. Ala2074Ter) is likely a novel genetic mutation type associated with palmoplantar keratoderma.

**Part II: Construction of Stable *DSP* Gene-Interference Cell Line**

**1. Materials and Methods**

**1.1 Experimental Materials**

1.1.1 Strains

Strain: Escherichia coli strain DH5α, used for auxiliary packaging of vector plasmids and amplification of lentiviral vectors.

1.1.2 Cell Lines

HaCaT cells: Purchased from Shanghai Fuheng Biotechnology Co., Ltd.

Packaging Cell Line: 293T cells, an adherent, epithelial-like cell line.

1.1.3 Lentiviruses

Control virus: HBLV-ZSGREEN-PURO.

Target viruses: HBLV-h-*DSP*-shRNA1-ZSGREEN-PURO, HBLV-h-*DSP*-shRNA2-ZSGREEN-PURO, HBLV-h-*DSP*-shRNA3-ZSGREEN-PURO.

1.1.4 Lentiviral Packaging System

The three-plasmid system: pSPAX2, pMD2.G, and the shuttle plasmid (carrying the target gene or shRNA).

1.1.5 Major Experimental Reagents

1.1.5.1 Cell Culture-Related Reagents

DMEM high-glucose medium (Gibco, C11995500BT); Trypsin’HAKATA ® Fetal; Bovine Serum (HN-FBS-500); Penicillin-Streptomycin (10,000 U/mL; Gibco, 15140122); PBS Buffer (Solarbio)

1.1.5.2 Lentiviral Vector Construction Reagents

| Reagent Name | Manufacturer |
| --- | --- |
| vector | Hanbio Biotechnology |
| DH5α competent cell | TIANGEN |
| phanta Max-Super-Fidlity DNA polymerase | Vazyme |
| HB-infusion^TM^ | Hanbio Biotechnology |
| Plasmid DNA purification | MACHEREY-NAGEL |
| Gel DNA purification | Generay |
| DNA laPPKer | Generay |
| Restriction Endonuclease | Thermo Scientific |

1.1.5.3 Lentiviral Packaging Reagents

| Reagent Name | Manufacturer | Catalog Number |
| --- | --- | --- |
| HB-Lipofiter™ | Hanbio Biotechnology | TRCF-1000 |
| PBS | Thermo Fisher | 10010001 |

1.1.5.4 Reagents for Generating Stable *DSP*-Knockdown HaCaT Cell Line

| Reagent/Consumable Name | Manufacturer | Catalog Number |
| --- | --- | --- |
| Fetal Bovine Serum | Gibco | 10091-148 |
| Trypsin | Gibco | 25200072 |
| DMEM | Gibco | C11995500BT |
| PBS | Thermo Fisher | 10010001 |
| Polybrene | Sigma | H9268 |
| Puromycin | Gibco | A1113802 |
| 50 mL Centrifuge Tube | NEST | 602002 |
| 15 mL Centrifuge Tube | NEST | 601002 |
| 5 mL Pipette | NEST | 326001 |
| 6-well Plate | Corning | 3516 |
| 60 mm Dish | Corning | 430166 |
| 100 mm Dish | Corning | 430167 |

1.1.5.5 qPCR-Related Reagents

| Reagent Name | Manufacturer | Catalog Number |
| --- | --- | --- |
| TRIzol Reagent | Thermo Fisher | 15596018 |
| Chloroform | Sigma | 151858 |
| Isopropanol | Sigma | W292907 |
| Ethanol, Absolute | Sigma | 459836 |
| DEPC Water | Thermo Fisher | AM9906 |
| Agarose | Sigma | A600014 |
| ReverTra Ace® qPCR RT Kit | TOYOBO | FSQ-301 |
| SYBR Green Realtime PCR Master Mix | TOYOBO | QPK-201 |
| Forward/Reverse Primers | Shanghai Biosune Biotechnology | - |

-

1.1.6 Major Laboratory Instruments

| Instrument Name | Manufacturer | Model/Catalog Number |
| --- | --- | --- |
| PCR System | BIO-Rad | T100 |
| 384-well PCR System | Shanghai Qibu | WSB-2P-384 |
| Centrifuge | Xiangyi | TGL-16 |
| 37°C Constant Temperature Incubator | Jinghong | GNP 9050 |
| Constant Temperature Water Bath | Yiheng | HSW S26 |
| Clean Bench | Sujing | SW-CJ-1B |
| UV Imaging System | Tanon | Tanon 1200 |
| Nucleic Acid Electrophoresis System | Tanon | EPS 300 |
| Horizontal Electrophoresis Tank | Tanon | HE-120 |
| Cell Culture Incubator | Thermo | 3111 |
| Biosafety Cabinet | Labconco | DELTA A2 |
| High-Speed Refrigerated Centrifuge | Thermo | ST 40 R |
| Inverted Biological Microscope | Olympus | CKX53 |
| Ultra-Speed Freezing Centrifuge | Hitachi | CP-100WX |
| High-Pressure Sterilizer | Shanghai Shen'an Medical Equipment Factory | LDZX-50KBS |

**1.2 Experimental Methods**

**1.2.1 Cell Culture**

1.2.1.1 Cell Thawing

Pre-set the water bath temperature to 37°C.

Retrieve the cryopreserved cells from the liquid nitrogen tank and immediately place them in a foam box containing dry ice or liquid nitrogen. Then, briefly place the cryovial in the water bath, gently agitate it, and remove it. Repeat this process once more. After confirming no abnormalities with the cryovial, immerse it fully in the water bath and shake it vigorously to ensure even thawing. The thawing process should be completed within 1 minute to minimize the formation of damaging ice crystals. Avoid thawing too many vials simultaneously, as this can reduce heat transfer efficiency and prolong the thawing time.

Prepare a 10 mL or 15 mL centrifuge tube pre-filled with an equal volume of fresh complete medium. Transfer the thawed cell suspension into this tube. Mix gently or resuspend carefully 1-2 times. Centrifuge at 1000 rpm for 5 minutes.

Discard the supernatant. Add 1 mL of fresh complete medium and gently resuspend the cell pellet approximately 15 times. Transfer the suspension to a culture flask or dish. Add additional medium and mix gently, for example, using a figure-eight motion.

Place the culture flask or dish steadily into the incubator. Maintain culture conditions at 37°C, 5% CO₂, and 95% relative humidity.

Observe cell attachment after 4-6 hours. Check cell growth the next day and replace the medium. Monitor cell growth daily and passage the cells when they reach approximately 80% confluence.

1.2.1.2 Cell Passaging

Passage cells when they reach approximately 80% confluence. Discard the old medium and wash the cell layer twice with sterile PBS. Discard the wash solution.

Add an appropriate volume of 0.25% trypsin-EDTA solution (e.g., 2 mL for a 10 cm dish) and incubate in the incubator for 4-5 minutes. Observe under an inverted microscope until cell gaps increase and cells become rounded. Immediately add an equal volume of the appropriate complete medium to stop trypsinization.

Gently pipette repeatedly to detach the adherent cells and create a single-cell suspension. Transfer the suspension to a 15 mL centrifuge tube.

Centrifuge at 1000 rpm for 3-5 minutes. Discard the supernatant.

Resuspend the cell pellet in fresh complete medium. Based on the recommended subcultivation ratio for HaCaT cells (1:3), aliquot the cell suspension into new culture flasks or dishes. Add sufficient fresh complete medium. Place the cultures steadily in the incubator at 37°C, 5% CO₂, and 95% relative humidity. Observe cell attachment status after 4-6 hours.

1.2.1.3 Cell Cryopreservation

Harvest logarithmically growing cells. Discard the supernatant, wash with PBS, and discard the wash. Add an appropriate volume of trypsin, incubate until cells detach, then add an equal volume of complete medium to neutralize the trypsin. Gently pipette to create a single-cell suspension and transfer it to a 15 mL centrifuge tube.

Centrifuge at 1000 rpm for 3-5 minutes. Discard the supernatant. Add an appropriate volume of cell freezing medium and resuspend the cell pellet gently by pipetting approximately 15 times. Typically, cells from one confluent 10 cm dish (≥90% confluence) are aliquoted into two cryovials.

Transfer the cell suspension in freezing medium into cryovials. Label each vial clearly with the cell line, gene modification, operator's name, and date. Use a controlled-rate freezing container or a stepwise cooling protocol to gradually lower the temperature. Finally, store the vials at -80°C overnight. For long-term storage, transfer the vials to a liquid nitrogen tank.

1.2.2 Construction of the Interference Lentiviral Vector

The interference vector used was: pHBLV-U6-MCS-CMV-ZsGreen-PGK-PURO.


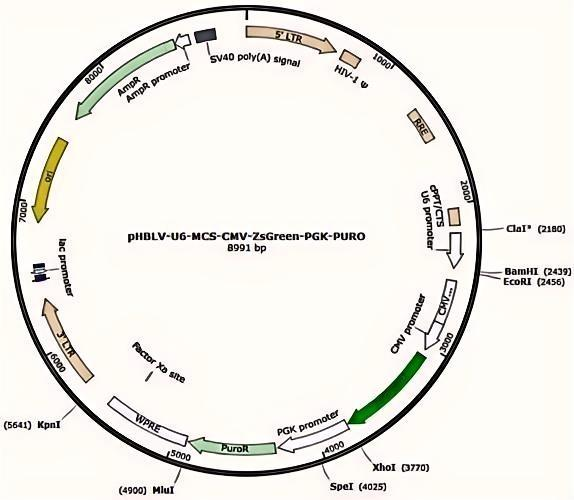


1.2.2.1 Interference Target Design and Primer Synthesis
The sequences for the control viral vector siRNA and shRNA are as follows:

siRNA sequence: TTCTCCGAACGTGTCACGTAA

shRNA sequence:

Top strand:

GATCCGTTCTCCGAACGTGTCACGTAATTCAAGAGATTACGTGAC

ACGTTCGGAGAATTTTTTC

Bottom strand:

AATTGAAAAAATTCTCCGAACGTGTCACGTAATCTCTTGAATTAC

GTGACACGTTCGGAGAACG

The siRNA and shRNA sequences for the target gene are:

siRNA1 sequence: CCTGAATTGAAGTATGGAGAT

shRNA1 sequence:

Top strand:

GATCCGCCTGAATTGAAGTATGGAGATCTCGAGATCTCCATACTT

CAATTCAGGTTTTTTG

Bottom strand:

AATTCAAAAAACCTGAATTGAAGTATGGAGATCTCGAGATCTCC

ATACTTCAATTCAGGCG

siRNA2 sequence: AGCAAATGCGAGCCCTTTATA

shRNA2 sequence:

Top strand:

GATCCGAGCAAATGCGAGCCCTTTATACTCGAGTATAAAGGGCT

CGCATTTGCTTTTTTTG

Bottom strand:

AATTCAAAAAAAGCAAATGCGAGCCCTTTATACTCGAGTATAAAGGG

CTCGCATTTGCTCG

siRNA3 sequence: CCAGACTACAGAAGCAATAAA

shRNA3 sequence:

Top strand:

GATCCGCCAGACTACAGAAGCAATAAACTCGAGTTTATTGCTTCT

GTAGTCTGGTTTTTTG

Bottom strand:

AATTCAAAAAACCAGACTACAGAAGCAATAAACTCGAGTTTATT

GCTTCTGTAGTCTGGCG

1.2.2.2 Primer Annealing to Form Double-Stranded Fragments with Sticky Ends
Dilute the primers to 100 μM each. The annealing reaction mixture (20 μL) is prepared as follows: add 1 μL each of the forward and reverse primers, 2 μL of 10× oligo Annealing Buffer, and then add H₂O to a final volume of 20 μL.
The annealing program is: 95°C for 10 min, 75°C for 10 min, 55°C for 10 min, 35°C for 10 min, and 15°C for 10 min.

1.2.2.3 Vector Digestion
The vector digestion reaction mixture (40 μL) is prepared as follows: 4 μL of 10× restriction enzyme buffer, 1.5 μL each of Restriction Enzyme 1 and 2, 1 μL of vector DNA (1 μg/μL), and then add PPK H₂O to a final volume of 40 μL. Mix the reagents gently.
Incubate the reaction mixture in a 37°C water bath for 1-2 hours. After digestion, perform electrophoresis and excise the gel to recover the target DNA fragment.

1.2.2.4 Ligation of Interference Fragment and Vector
The ligation reaction mixture (20 μL) is prepared as follows: 2 μL of T4 DNA Ligase Buffer, 1 μL of T4 DNA Ligase, 4 μL of the annealed product, X μL of the digested vector (≥50 ng), and then add H₂O to a final volume of 20 μL.
Incubate the reaction mixture at 22°C for 1-2 hours, or at 16°C overnight.

1.2.2.5 Transformation

1. Thaw DH5α competent cells on ice. After thawing, aliquot 50 μL per tube. Handle gently to avoid mechanical damage.
2. Add 5 μL of the ligation product to the competent cells and incubate on ice for 20-30 minutes.
3. Heat-shock the cells at 42°C for 90 seconds, then immediately place them on ice for 2-3 minutes.
4. Transfer the tubes to a biosafety cabinet, add 500 μL of LB medium, and gently invert the tubes 3-5 times.
5. Incubate the tubes in a shaker incubator at 37°C, 230 rpm, for 50 minutes.
6. Spread the bacterial culture evenly onto solid agar plates containing the appropriate antibiotic. Invert the plates and incubate at 37°C for 12-16 hours.

#### Bacterial Colony PCR Identification

1). The bacterial colony PCR reaction mixture (10 μL) is prepared as follows: 2 μL of bacterial culture, 5 μL of 2× Hieff PCR Master Mix (Dye), 0.5 μL each of Primer 1 and Primer 2, and 2 μL of PPK H₂O to a final volume of 10 μL.

2). Bacterial colony PCR program:

| Step | Temperature | Time | Cycles |
| --- | --- | --- | --- |
| Pre-Denaturation | 94°C | 5 min | 1 |
| Denaturation | 94°C | 30 sec |  |
| Annealing | 56°C | 30 sec | 25 |
| Extension | 72°C | 30-60 sec/kb |  |
| Final Extension | 72°C | 10 min | 1 |
| Hold | 12°C | ∞ | 1 |

Sequence analysis of the three constructed plasmids confirmed that the sequencing results were consistent with the target sequences, indicating the successful construction of the target plasmids. The sequencing results are as follows:

h-*DSP*-shRNA1 Sequencing Result:


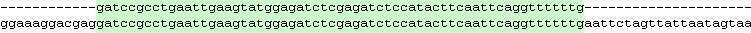


h-*DSP*-shRNA2 Sequencing Result:


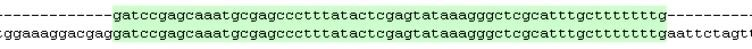


h-*DSP*-shRNA3 Sequencing Result:


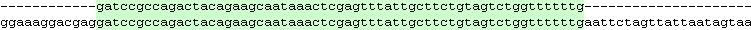


1.2.3 Lentiviral Packaging by Cell Transfection

1). Culture 293T cells at 37°C with 5% CO₂. When the cells are in good condition and reach 70-80% confluence, they are ready for transfection.

2). Prepare the lipid-transfection complex according to the Lipofiter™ manufacturer's instructions. For example, the transfection system for a 10 cm dish is as follows:

| Plasmid/Reagent | Amount |
| --- | --- |
| pMD2.G | 5 μg |
| pSPAX2 | 10 μg |
| Lipofiter™ | 75 μL |
| Shuttle Plasmid | 10 μg |

3). Add the reagents listed in the table above sequentially into a microcentrifuge tube. Mix gently and incubate at room temperature for 15 minutes to form the transfection complex. Then, add the complex dropwise slowly to the 293T cells. After addition, return the cells to the incubator.
4). 16 hours post-transfection, replace the medium with fresh culture medium.
5). Collect the viral supernatant at 48 hours and 72 hours post-transfection. Pour the medium from the culture dishes into 50 mL centrifuge tubes. Centrifuge at 4°C, 2000 × g for 10 minutes. Transfer the supernatant to ultracentrifuge tubes. Perform ultracentrifugation at 4°C, 82,700 × g for 120 minutes.
6). Discard the supernatant and resuspend the viral pellet in complete medium. Aliquot the virus suspension into sterilized cryovials. Label the vials with the virus name, date, etc., and store at -80°C.

1.2.4 Generation of Stable *DSP*-Knockdown HaCaT Cell Lines

1. Cell Seeding (Day 1)
   Seed HaCaT cells in good growth condition into 6-well plates, adding 2 mL of fresh medium per well.
2. Viral Infection (Day 2)
   Discard the medium from the wells. Add 2 mL of fresh medium containing 5% FBS. Then, add the appropriate amount of the transduction enhancer polybrene (final concentration used in this experiment: 7 μg/mL). Finally, add the calculated volume of viral supernatant.
   Virus volume calculation: (Cell number × Desired MOI / Viral stock titer) × 10³ = Virus volume (μL).
   Experimental Groups: Viruses were tested at MOI values of 5, 10, and 15, as outlined below:
   *(A table would be inserted here showing the groups: lv-PURO, lv-*DSP*-shRNA1-PURO, lv-*DSP*-shRNA2-PURO, lv-*DSP*-shRNA3-PURO, each at MOI=5, 10, 15)*
   (MOI, Multiplicity of Infection: Ratio of viral particles to target cells).
3. Medium Change (Day 3)
   24 hours after infection, discard the virus-containing medium and replace it with fresh medium.
4. Observation (Day 4)
   48 hours post-infection, observe cell status. For the *DSP*-knockdown groups, the MOI=15 condition was selected for all three shRNAs for subsequent experiments based on comparative assessment. In the negative control group, cell status was good across all MOI values, and the MOI=5 condition was ultimately selected for subsequent experiments.
5. Selection of Stable Cell Lines (Day 5)
   Prepare cell suspensions and seed them in 6-well plates in advance, aiming for ~60% confluence the next day. After 24 hours, replace the medium with medium containing a gradient of puromycin concentrations. After 48 hours of treatment, observe cell death. Select the lowest puromycin concentration that kills all wild-type HaCaT cells. In this experiment, the minimum puromycin concentration that killed wild-type HaCaT cells was 1.5 μg/mL.
6. Antibiotic Selection, Culture, and Verification
   Continue culturing the cells with fresh medium containing 1.5 μg/mL puromycin. After successful selection for two passages with good cell survival, passage the cells at an appropriate ratio. For subsequent culture after thawing, maintain the cells in medium containing 1.0 μg/mL puromycin, changing the medium every 2-3 days. Once the cell growth is stable, harvest samples for RNA extraction and QPCR verification.

1.2.5 QPCR Verification of Knockdown Efficiency

1.2.5.1 Total RNA Extraction from Virus-Infected Cells

1. Seed cells in a 6-well plate. When cells reach full confluence, discard the supernatant and add 1 mL of TRIzol Reagent per well. Mix thoroughly to lyse the cells.
2. Transfer the lysate to a 1.5 mL microcentrifuge tube and incubate at room temperature for 10 minutes.
3. Add 200 μL of chloroform, vortex vigorously, and incubate at room temperature for 10 minutes.
4. Centrifuge at 4°C, 13,780 × g for 10 minutes. Transfer the upper aqueous phase to a new tube. Add an equal volume of isopropanol to the new tube and incubate at room temperature for 10 minutes.
5. Centrifuge at 4°C, 13,780 × g for 15 minutes. Discard the supernatant.
6. Wash the pellet once with 1 mL of 75% ethanol. Centrifuge at 4°C, 13,780 × g for 5 minutes. Discard the supernatant and keep the pellet. Air-dry the pellet inverted at room temperature for about 10 minutes.
7. Dissolve the RNA pellet in DEPC-treated water. Measure the OD260 and OD280 values and calculate the RNA concentration.

1.2.5.2 Reverse Transcription of RNA into cDNA
Perform reverse transcription according to the reverse transcription kit instructions and operational manual to synthesize cDNA. The cDNA can be stored at -20°C for subsequent PCR detection.

1.2.5.3 qPCR Primer Design
Primers for qPCR were designed based on the target gene sequences and synthesized. The primer sequences are as follows:

| Primer Name | Primer Sequence (5' to 3') |
| --- | --- |
| h-*DSP*-Q-F | CTCCAAGAGCAAATGCGAGC |
| h-*DSP*-Q-R | ATGTCCATCTCCGCCCTTTG |
| h*GAPDH*-Q-F | TCAAGGCTGAGAACGGGAAG |
| h*GAPDH*-Q-R | TCGCCCCACTTGATTTTGGA |

**1.2.5.4 Real-time PCR Reaction Setup and Conditions**
The Real-time PCR reaction mixture is as follows: 1 μL cDNA Template (1:100 dilution), 5 μL of 2× Real-time PCR Master Mix, 0.4 μL each of Forward and Reverse Primer (10 μM), and PPK H₂O is added to a final volume of 10 μL.
The reaction conditions are: 95°C for 180 s; 40 cycles of: 94°C for 15 sec, 60°C for 30 sec, 72°C for 30 sec; followed by: 95°C for 10 sec, 65°C for 60 sec, 97°C for 1 sec.

**2.Result**

**2.1 Construction of the HaCaT-h-*DSP*-shRNA-ZSGREEN-PURO Cell Line**

This experiment successfully established the target cell line with stable *DSP* knockdown (HaCaT-h-*DSP*-shRNA-ZSGREEN-PURO) and the control cell line (ZSGREEN-PURO) in the HaCaT cell line. After passaging, when the cells reached full confluence, samples were harvested for qPCR analysis. The results indicated that the shRNA2 (sh2) group exhibited satisfactory knockdown efficiency. Consequently, cells from the sh2 group were selected for large-scale expansion and cryopreservation. The cryopreserved stable *DSP*-knockdown cells and control cells were subsequently thawed. Microscopic examination confirmed good cell morphology, absence of contamination, and clear expression of green fluorescence.

Growth status of interfering *DSP* stable strain and control cell lines

| Group | Bright Field | Fluorescence |
| --- | --- | --- |
| HaCaT-ZSGREEN-PURO | 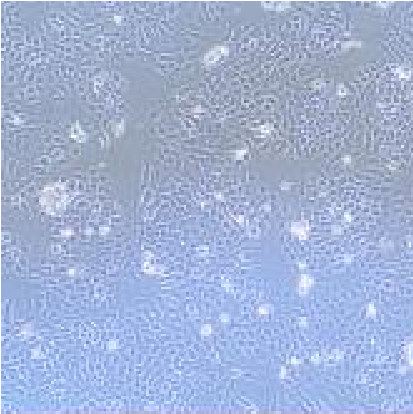 | 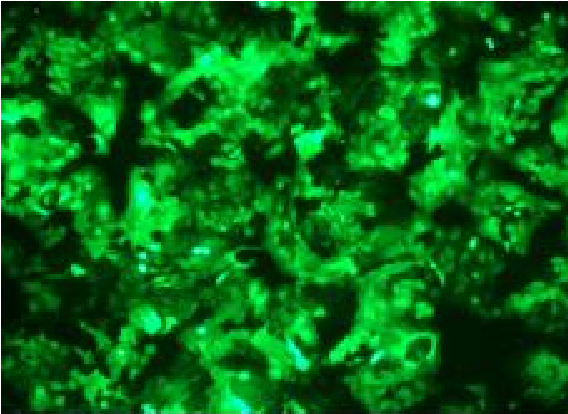 |
| HaCaT-h-*DSP*-shRNA2-ZSGREEN-PURO | 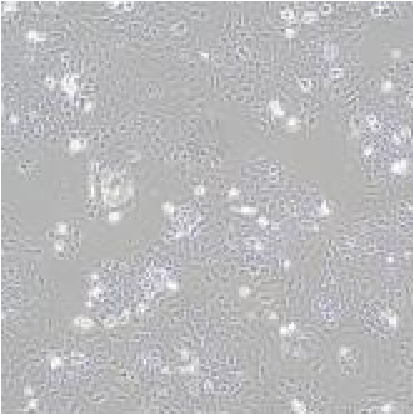 | 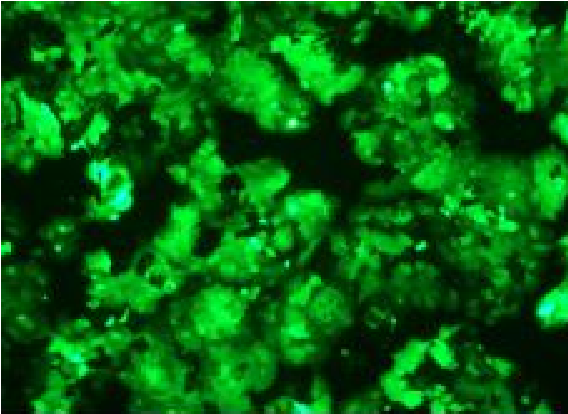 |

**2.2 Screening for the Optimal Target in the HaCaT-h-*DSP*-shRNA-ZSGREEN-PURO Cell Line**

This experiment aimed to screen for the optimal knockdown efficiency in the HaCaT-h-*DSP*-shRNA-ZSGREEN-PURO cell line by assessing the relative mRNA expression levels of the reference gene *GAPDH* and the target gene *DSP* in HaCaT-NC, h-*DSP*-sh1, h-*DSP*-sh2, and h-*DSP*-sh3 cells using conventional PCR-gel electrophoresis and qPCR techniques.

UV spectrophotometry analysis of the extracted RNA quality showed that the OD260/OD280 and OD260/OD230 ratios for all groups fell within the acceptable reference ranges (OD260/OD280: 1.8-2.4; OD260/OD230: 1.5-2.4), indicating satisfactory RNA purity (Supplementary Table 6). RNA agarose gel electrophoresis analysis revealed no obvious differences in band integrity between the groups upon visual inspection (Figure 6). qPCR results demonstrated clean dissociation curves for both *GAPDH* and *h-DSP*, confirming the absence of significant issues during RNA extraction and the qPCR process (Figure 7). Compared to the HaCaT-NC cells, the h-*DSP*-sh2 group exhibited a knockdown efficiency of 51%. Furthermore, shRNA2 (sh-2) demonstrated the highest interference efficiency among the three different target sites tested (Figure 3).

(1) RNA Quality Assessment Results

Supplementary Table 6 RNA Quality Assessment Results.

| Sample Name | OD260/OD280 | OD260/OD230 | RNA Concentration (ng/μL) |
| --- | --- | --- | --- |
| HaCaT-NC | 1.96 | 2.06 | 537 |
| h-*DSP*-Sh1 | 1.95 | 2.04 | 529 |
| h-*DSP*-Sh2 | 1.92 | 2.02 | 542 |
| h-*DSP*-Sh3 | 1.93 | 2.01 | 537 |

(2) RNA Agarose Gel Electrophoresis Analysis Results (Supplementary Figure 3):


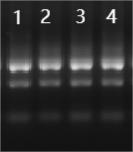


Supplementary Figure 3 Results of RNA agarose gel electrophoresis analysis

（Note：1: HACAT-NC; 2: h-*DSP*-sh1；3: h-*DSP*-sh2;4: h-*DSP*-sh3）

(3). Dissociation curves for *GAPDH* and h-*DSP* genes are shown


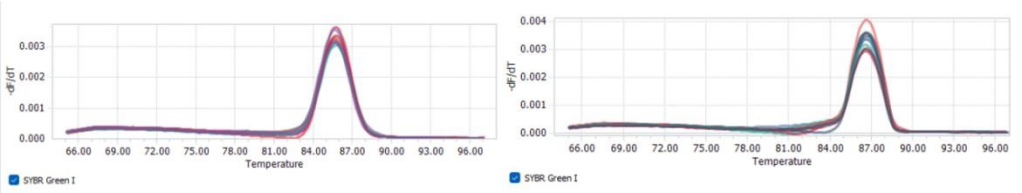


Supplementary Figure 4 Lysis curves of *GAPDH* and *h-DSP* genes

*GAPDH* gene lysis curve (left); h-*DSP* gene lysis curve (right).

(4) Relative *DSP* Expression Level: Compared to the HaCaT-NC cells, the h-*DSP*-sh2 group exhibited a knockdown efficiency of 51%. Furthermore, shRNA-2 (sh-2) demonstrated the highest interference efficiency among the three different shRNA targets tested.


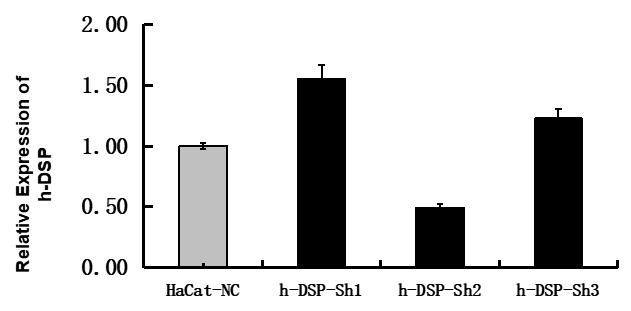


Supplementary Figure 5 Evaluation of the effect of three *DSP* gene interference targets.

**3. Conclusion**

This study successfully constructed a stable *DSP* knockdown cell line, namely the HaCaT-h-*DSP*-shRNA-ZSGREEN-PURO cell line, utilizing the pHBLV-U6-MCS-CMV-ZsGreen-PGK-PURO lentiviral vector system. This work establishes a fundamental and reliable experimental resource for subsequent cellular-level research involving *DSP*-knockdown in HaCaT cells.

**Part III: Effect of *DSP* on HaCaT Cell Adhesion and Proliferation**

**1. Materials and Methods**

**1.1 Experimental Materials**

1.1.1 Cells

Stable *DSP*-knockdown cell line: HaCaT-*DSP*-shRNA2-ZSGREEN-PURO

Negative control cell lines: HaCaT-ZSGREEN-PURO, HaCaT

HaCaT cells: Purchased from Shanghai Fuheng Biotechnology Co., Ltd.

1.1.2 Major Kits and Enzymes

Cell Adhesion Assay Kit was purchased from Shanghai BB Biotechnology Co., Ltd. (Catalog No.: BB-48120).

CCK-8 Assay Kit was purchased from Beyotime Biotechnology (Catalog No: C0038).

1.1.3 Major Laboratory Instruments

| Instrument Name | Manufacturer |
| --- | --- |
| 37°C Constant Temperature Incubator | Jinghong |
| Constant Temperature Water Bath | Yiheng |
| Clean Bench | Sujing |
| Micropipettes | Thermo |
| Nucleic Acid Electrophoresis System | Tanon |
| Microplate Reader | Thermo |
| High-Pressure Sterilizer | Shanghai Shen'an Medical Equipment Factory |
| Inverted Biological Microscope | Olympus |
| 4°C Benchtop Centrifuge | Eppendorf |
| Room Temperature Benchtop Centrifuge | Thermo |
| Cell Culture Flasks/Dishes/Plates | Corning |
| Centrifuge Tubes | Corning |
| Pipette Tips | Corning |
| Cryovials | Corning |
| Microcentrifuge Tubes | Axygen |

**1.2 Experimental Methods**

1.2.1 Cell Adhesion Assay

1.2.1.1 Coating

1. Add 100 μL of coating solution to each well of a 96-well plate.
2. Incubate the plate at 2-8°C overnight.
3. Remove the coating solution.
4. Dry the plate. Air-dry for several minutes at room temperature in a ventilation hood until completely dry visually.
5. Wash three times with the Wash Buffer provided in the kit.

1.2.1.2 Cell Seeding

1. Harvest the test cells by trypsinization, resuspend, and prepare a cell suspension.
2. Seed the cells into the 96-well plate at a density of 5 × 10⁴ cells/well, with 3-5 replicate wells per group.
3. Incubate the plate at 37°C for 1 hour.
4. Remove the plate, aspirate the medium, and wash the wells twice with the same medium.
5. Add 100 μL of fresh medium to each well.

1.2.1.3 Adhesion Rate Detection

1. Add 10 μL of Cell Staining Solution B to each well of the 96-well plate.
2. Incubate the plate in the incubator for 2 hours.
3. Measure the OD value at 450 nm using a microplate reader.
4. Calculate the cell adhesion rate: Cell Adhesion Rate % = [(OD_ sample - OD_ blank) / (OD_ control - OD_ blank)] × 100%. Use the average OD value of the replicate wells.

1.2.2 CCK-8 Assay for Cell Proliferation

1. Prepare a cell suspension and count the cells.
2. Seed the cell suspension into 96-well plates, approximately 100 μL per well, with about 2000 cells/well. Set up 5 replicate wells per group and time point. Seed cells into four 96-well plates.
3. Culture the plates for 24 h, 48 h, 72 h, and 96 h after seeding.
4. At each time point, add 10 μL of CCK-8 solution to each well (avoiding bubbles), mix gently, and return the plate to the incubator for an additional 2 hours (the incubation time can be optimized in preliminary experiments).
5. Measure the absorbance (OD) at 450 nm for each group using a microplate reader.

**1.3 Statistical Analysis**
Data were processed using SPSS 26.0 statistical software. Data are expressed as mean ± standard deviation (x ± s). Differences between two groups were analyzed using the independent samples t-test. A *P-value < 0.05* was considered statistically significant.

**2 Results**

**2.1 Effect of DSP on Keratinocyte Cell Adhesion Rate**
The OD values at 450 nm were measured using a cell adhesion assay kit and a microplate reader. The results showed a significant difference between the sh*DSP* group and the NC group (*P < 0.05*) (see Table 2). A bar graph was plotted with the group as the X-axis and the OD reading at 450 nm as the Y-axis, illustrating the OD values for the sh*DSP* and NC groups. The cell adhesion rate of HaCaT cells after *DSP* knockdown was calculated as (OD_ sh*DSP* / OD_NC) × 100%, resulting in 53.82%.

These results indicate that the cell adhesion ability of HaCaT- sh*DSP* cells was significantly weaker than that of HaCaT cells, suggesting that *DSP* knockdown impairs the cell adhesion ability of keratinocytes.

Supplementary Table 7 Detection of cell adhesion rate of HaCaT cells after *DSP* interference (x ± s)

| Group | OD Value |
| --- | --- |
| HaCaT-NC Group | 0.959 ± 0.064 |
| HaCaT-shDSP Group | 0.516 ± 0.060 |
| *F-value* | 0.010 |
| *P-value* | 0.000 |

Comparison between sh*DSP* group and NC group, *P* < 0.05.

**2.2 Effect of *DSP* on Keratinocyte Cell Proliferation Capacity**

The OD values at 450 nm were measured using the CCK-8 kit and a microplate reader. The results showed a significant difference between the sh*DSP* group and the NC group (*P < 0.05*) (see Table 3). A proliferation curve for the two cell lines was plotted with time (days) as the X-axis and the OD reading at 450 nm as the Y-axis. Over the 4-day period, the cell numbers in both groups increased with time, but the proliferation of the stable *DSP*-knockdown cell line was significantly inhibited.

These cell proliferation experiment results indicate that the proliferation capacity of HaCaT-shDSP cells is weaker than that of HaCaT-NC cells, suggesting that *DSP* knockdown can reduce the proliferation ability of keratinocytes.

**Table 3** Detection of cell proliferation of HaCaT cell line after *DSP* interference (x ± s)

| Group | OD Value (Day 1) | OD Value (Day 2) | OD Value (Day 3) | OD Value (Day 4) |
| --- | --- | --- | --- | --- |
| NC Group | 0.100 ± 0.002 | 0.201 ± 0.025 | 0.368 ± 0.074 | 0.550 ± 0.098 |
| sh*DSP* Group | 0.073 ± 0.008 | 0.156 ± 0.059 | 0.191 ± 0.023 | 0.260 ± 0.041 |
| *F-value* | 19.786 | 1.942 | 26.657 | 1.052 |
| *P-value* | 0.001 | 0.150 | 0.004 | 0.000 |

**4 Conclusion**

Knockdown of *DSP* led to reduced cell adhesion and proliferation capacities in keratinocytes. At the cellular level, this verifies that *DSP* expression is associated with the cell adhesion function of keratinocytes.
